# Efficacy and Safety of Acupuncture for Post–COVID-19 Insomnia: Protocol for a Systematic Review and Meta-Analysis

**DOI:** 10.2196/69417

**Published:** 2025-03-03

**Authors:** Yadi Li, Jianlong Zhou, Zheng Wei, Lizhu Liang, Hualing Xu, Caihong Lv, Gang Liu, Wenlin Li, Xin Wu, Yunhui Xiao, Kejimu Sunzi

**Affiliations:** 1 Deyang People's Hospital Deyang China; 2 Department of Neurology Chengdu University of Traditional Chinese Medicine Chengdu China; 3 Department of Endocrinology Chengdu University of Traditional Chinese Medicine Chengdu China

**Keywords:** acupuncture, traditional Chinese medicine, post–COVID-19 condition, long COVID-19, insomnia, sleep disorder, depression, complementary and alternative medicine, treatment, public health, study protocol, systematic review

## Abstract

**Background:**

The COVID-19 pandemic has had a profound global impact, leading to a range of persistent sequelae referred to as post–COVID-19 condition or “long COVID” that continue to affect patients worldwide. Among these sequelae, post–COVID-19 insomnia (PCI) has emerged as a significant issue. Conventional treatments, including cognitive behavioral therapy and pharmacological interventions, face limitations such as variable efficacy, potential side effects, and substantial costs. Recently, acupuncture has gained traction due to its efficacy, cost-effectiveness, and safety profile.

**Objective:**

This study aims to conduct a meta-analysis and systematic review evaluating the efficacy and safety of acupuncture for the treatment of PCI to delineate the optimal modality, intervention frequency, and duration for achieving the most beneficial outcomes, thereby providing a comprehensive understanding of acupuncture’s role in managing PCI, contributing to evidence-based clinical practice, and informing clinical decision-making.

**Methods:**

Electronic searches will be performed in 12 databases from inception to October 2024 without language restrictions. This includes both English databases (PubMed, Cochrane Library, Web of Science, Embase, OVID and Scopus), as well as Chinese databases (China National Knowledge Infrastructure, Wan-Fang Data, Chinese Biomedical Literature Database, Chinese Scientific Journal Database, Duxiu Database and the Chinese Clinical Trial Registry Center). Randomized controlled trials on acupuncture for PCI will be included. Primary outcomes will include the response rate and insomnia severity; secondary outcomes will include the Traditional Chinese Medicine Symptom Scale (TCMSS) and adverse event rates. Data synthesis will use risk ratios for dichotomous data and mean differences for continuous data. Study selection, data extraction, and quality assessment will be conducted independently by 2 reviewers. Methodological quality of eligible studies will be evaluated following the *Cochrane Handbook for Systematic Reviews of Interventions* (version 6.3). Meta-analysis will be performed with RevMan 5.3.

**Results:**

Based on the data on response rate, insomnia severity, TCMSS score, and adverse event rates, this study will provide an evidence-based review of the efficacy and safety of acupuncture for PCI treatment.

**Conclusions:**

This systematic review will present the current evidence for acupuncture for PCI, aiming to inform clinical practices and decision-making and to enhance the understanding of acupuncture’s role in managing PCI. Furthermore, it will identify research gaps and suggest potential areas for future investigation.

**Trial Registration:**

PROSPERO CRD42024499284; https://www.crd.york.ac.uk/prospero/display_record.php?RecordID=499284

**International Registered Report Identifier (IRRID):**

DERR1-10.2196/69417

## Introduction

### Background

The COVID-19 pandemic caused by the SARS-CoV-2 virus has emerged as one of the most significant public health challenges in recent years [1], with over 770 million cases and approximately 6.9 million deaths worldwide [2,3]. Despite the World Health Organization having declared the end of the global public health emergency in May 2023, evolving virus variants continue to impact the world, exacerbating the global effects of the pandemic [4]. Nearly 90% of hospitalized patients with COVID-19 develop post–COVID-19 condition [5,6], commonly referred to as “long COVID” according to the National Institute for Health and Care Excellence, which includes a range of signs and symptoms that continue or develop after the acute phase of the illness [7]. These symptoms include sleep disturbances, depression, fatigue, tinnitus, and anxiety [8,9].

Studies indicate that insomnia has a high prevalence, impacting roughly 20% to 35% of the general population [10-13], and this rate is even higher among health care workers and patients with COVID-19, reaching up to 75% [8]. Substantial evidence indicates that post–COVID-19 insomnia (PCI) frequently persists over the long term [14-17], leading to serious health issues such as increased depression, prolonged work absences, and a heightened risk of hypertension [15,18,19]. Insomnia poses a significant threat to public health, not only by impairing physical, emotional, and social well-being but also by reducing quality of life [20]. It can degrade psychological functioning and decision-making, compromise the immune system, raise the risk of accidents, and cause mood alterations, all while boosting medical expenditures [21,22]. Moreover, the interaction between sleep deprivation and SARS-CoV-2 infection could potentially raise the risk of developing neurodegenerative diseases like dementia [16]. Consequently, PCI has become widely acknowledged as a critical and pressing issue affecting the entire world. The emergence of SARS-CoV-2 subvariants in the postpandemic COVID-19 era, particularly the highly mutable Omicron lineage and its numerous subvariants, presents new challenges to public health interventions [23].

Current standard treatments for PCI include cognitive behavioral therapy for insomnia (CBT-i) and pharmacotherapy [24,25]. Although these methods have demonstrated efficacy, they are not without limitations. For instance, the 2017 clinical practice guidelines of the American Academy of Sleep Medicine indicate that CBT-i alone may not be beneficial for all people with insomnia due to issues like noncompliance, treatment unresponsiveness, access barriers, and financial constraints [25]. Pharmacological therapies for insomnia comprise benzodiazepines, nonbenzodiazepines, antidepressants, melatonin receptor agonists, and other sedatives [24,26]. While these medications can offer short-term relief, their long-term, routine use is not recommended due to the risks of developing tolerance, dependency, and withdrawal issues [20,26-28]. Moreover, these medications can lead to rebound insomnia, residual daytime sedation, cognitive and memory impairments, and motor incoordination, particularly increasing the risk of falls among older people [20,26-29]. Given these concerns, there is a pressing need to find alternative therapeutic approaches that are both efficacious and safe for treating PCI.

In recent years, complementary and alternative medicine (CAM) has become widely used to address insomnia in various countries, including the United States, Australia, and numerous Asian nations [30-32]. Acupuncture, recognized as one of the most popular and safest CAM therapies [28,33], has a long-standing history of treating sleep disorders dating back to ancient times [26]. Based on traditional Chinese medicine, acupuncture works by inserting fine needles into acupoints throughout the body. It seeks to reestablish the equilibrium between yin and yang, thereby facilitating the body’s return to a state of physiological homeostasis [34-36]. A multitude of studies indicate that acupuncture is highly effective in improving both sleep quantity and quality [37-42]. The underlying mechanisms include regulating neurotransmitters such as gamma-aminobutyric acid [43-46] and serotonin [47-50], increasing melatonin levels [51-53], and modulating the hypothalamic-pituitary-adrenal axis and the expression of corticotropin-releasing hormone and adreno­corticotropic hormone [54-57], thereby improving sleep quality. Furthermore, acupuncture can also regulate inflammatory cytokines such as interleukin-1, interleukin-6, and tumor necrosis factor-α [58-62], and it can influence the gut microbiota to regulate sleep-wake behavior [63,64].

Much of the research supporting the use of acupuncture for insomnia is constrained by the limitations of low- or moderate-quality evidence and inconsistent reports regarding its clinical efficacy and safety. Furthermore, the effects of acupuncture on PCI remain largely unknown. Through an extensive literature review, we identified a notable absence of systematic reviews specifically addressing the efficacy of acupuncture for PCI. In light of this gap, we will conduct a meta-analysis and systematic review to assess and synthesize the available evidence on the efficacy and safety of acupuncture for PCI. This review aims to establish a robust theoretical foundation for clinical practice, thereby providing credible evidence on the therapeutic potential of acupuncture for PCI.

### Objectives

This review has 3 objectives: (1) to evaluate the efficacy and safety of acupuncture for the treatment of PCI in comparison to comfort therapy (such as placebo, sham acupuncture, or a blank control) or other therapies (such as Western medicine or nondrug therapies); the results will provide a comprehensive understanding of acupuncture’s role in managing PCI, thereby contributing to evidence-based clinical practice and informing clinical decision-making in practice; (2) to assess the methodological quality and the strength of the evidence supporting the use of acupuncture for PCI, offering a thorough appraisal of the existing literature; and (3) to delineate the optimal modality, intervention frequency, and duration to achieve the most beneficial outcomes.

## Methods

### Overview

This study will be conducted following the Preferred Reporting Items for Systematic Reviews and Meta-Analyses Protocols (PRISMA) statement [65]. This protocol has been prepared in accordance with the Cochrane recommendations and registered on PROSPERO (CRD42024499284).

### Study Eligibility Criteria

The inclusion and exclusion criteria for this review are detailed in Textbox 1.

**Inclusion and exclusion criteria.**

**Inclusion criteria**

- Types of studies
  All randomized controlled trials (RCTs) focusing on the treatment of insomnia after COVID-19 using acupuncture therapy
  Studies involving single acupuncture therapy, as well as those comparing acupuncture with other interventions
  Studies published in English or Chinese
  Studies published from the inception of the database up to October 2024
  No restrictions on publication status or type
- Types of participants
  Participants who developed insomnia following COVID-19 infection without a prior history of insomnia who were subsequently enrolled in an RCT; this will include patients with confirmed COVID­19 or asymptomatic SARS­CoV-2 infections based on the results from nucleic acid testing, antigen testing, and clinical criteria
  Participants meeting the diagnostic criteria for insomnia, including the *Diagnostic and Statistical Manual of Mental Disorders, Fifth Edition* [66], *Diagnostic and Statistical Manual of Mental Disorders, Fourth Edition* [67], *International Classification of Sleep Disorders, Third Edition)* [68], and *Chinese Classification and Diagnostic Criteria of Mental Disorders, Third Edition* [69] or equivalent standard diagnostic criteria
  No restrictions on gender, age, race, onset time, or source of cases
- Types of intervention
  Simple acupuncture therapy
  Trials where acupuncture is used as an adjunct to other interventions (eg, behavior therapy, psychotherapy, or pharmacotherapy) only included if the control groups receive the same additional interventions
  No specific restrictions on acupoints or course of treatment; the selection of acupoints and acupuncture methods should be derived from traditional Chinese medicine
  The specific length, thickness, and model of the acupuncture needle will not be considered in this review
- Types of comparisons
  Comfort therapy (placebo, sham acupuncture, or blank control)
  Other therapies (Western medicine or nondrug therapy)
- Types of outcome measures
  The primary outcomes will include response rate and insomnia severity as measured by validated instruments (eg, the Insomnia Severity Index, Pittsburgh Sleep Quality Index, or Athens Insomnia Scale)
  The secondary outcomes will include the Traditional Chinese Medicine Syndrome Score Scale score and adverse events

**Exclusion criteria**

- Non-RCTs, such as reviews, animal experiments, commentary articles, letters, and case reports
- Participants with primary insomnia or a serious underlying disease that makes them unsuitable for acupuncture treatment
- Studies that explored interventions using nontraditional acupoints, such as ear acupuncture, scalp acupuncture, and other methods
- Duplicate publications
- Studies found in gray literature
- Studies with incomplete data

### Electronic Searches

A search strategy will be designed and conducted following the guidelines outlined in the *Cochrane Handbook*. The meta-analysis will be reported based on the PRISMA guidelines.

Electronic searches will be carried out by 2 independent researchers (YL and JZ) from initiation until October 2024. English databases (PubMed, Cochrane Library, Web of Science, Embase, OVID, Scopus) and Chinese databases (China National Knowledge Infrastructure, Wan-Fang Data, Chinese Biomedical Literature Database, Chinese Scientific Journal Database, and Duxiu Database) will be included in the search. The Chinese Clinical Trial Registry Center will also be searched for ongoing trials. There will be no restrictions on countries or publication types.

The search will use keywords such as *COVID-19*, *2019-nCoV infection*, *SARS-CoV-2 infection*, *acupuncture therapy*, *acupuncture treatment*, *pharmacoacupuncture treatment*, *sleep initiation and maintenance disorders*, *sleeplessness*, and *insomnia disorder*. To ensure a comprehensive search, a combination of subject headings (Medical Subject Headings) and free words will be used, regardless of the language or type of publication. The search will be conducted across all databases to identify all relevant articles. An example search strategy for the PubMed database is provided in Multimedia Appendix 1.

Similar search strategies will be used for the other databases. Additionally, in the case of studies for which the full text is not available, efforts will be made to contact the first author or corresponding author to obtain the necessary full text.

### Study Selection and Data Collection

#### Selection of Studies

**Figure 1:**
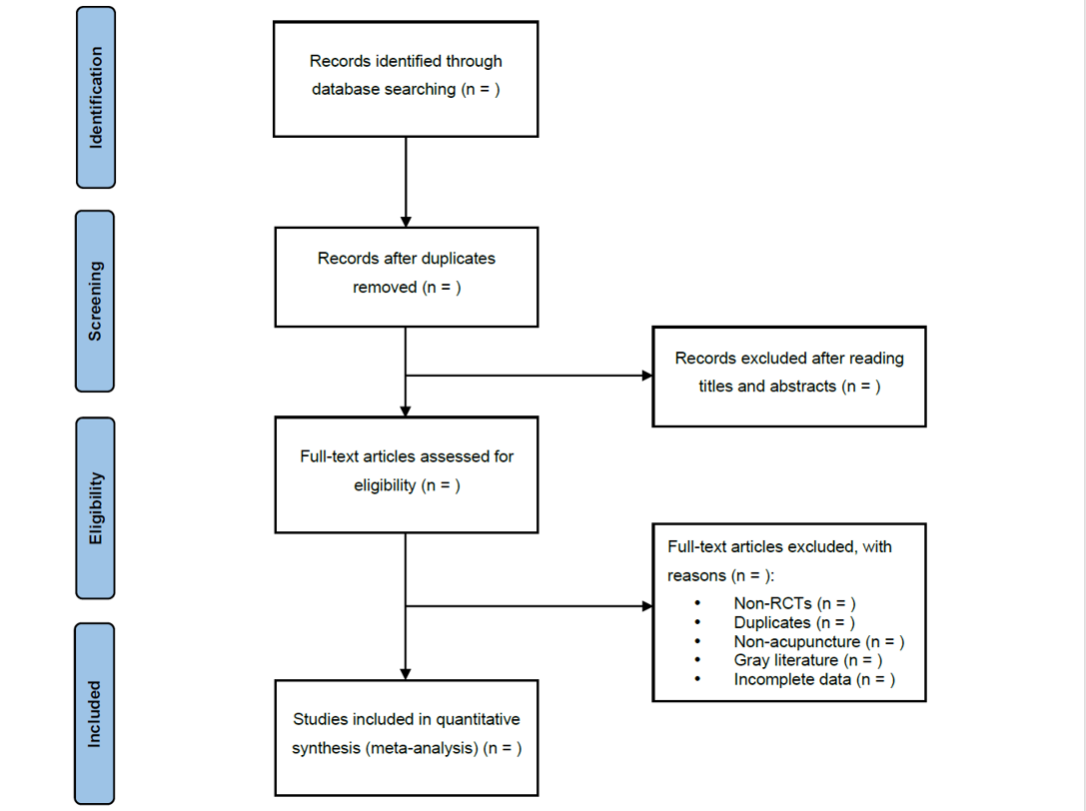
Preferred Reporting Items for Systematic Reviews and Meta-Analyses (PRISMA) flow chart. RCT: randomized controlled trial.

All reviewers have undergone training to ensure a fundamental comprehension of the context and purpose of the review. In this step, EndNote X9 (Clarivate) will be used to screen the search results. Two reviewers (YL and JZ) will independently conduct the screening process and cross-check the results. Based on the predefined inclusion and exclusion criteria, relevant studies will be selected after reviewing their titles, abstracts, or full texts. In instances of duplicate publications, the original publication will be selected. Should there be any incomplete data or ambiguous details, efforts will be made to contact the first author or corresponding author for clarification. In cases where disagreements arise, a third reviewer (KS) will be consulted, and a final decision will be reached through discussion. A comprehensive overview of the selection process will be depicted with a PRISMA flow chart (Figure 1).

#### Data Extraction and Management

Two reviewers (ZW and LL) will independently perform data extraction using the provided data extraction form (Multimedia Appendix 2). Any disagreements that emerge during the process will be addressed through discussion between the 2 reviewers. If a resolution cannot be achieved, the input of a third reviewer (HX) will be sought. The following information will be recorded using a standardized data extraction form: (1) basic information on the study (first author, year, and country of publication); (2) study characteristics (study inclusion criteria, sample size, and participants’ details, including age, gender, complications, severity, and course of insomnia); (3) details of the study intervention and control intervention, including modality, frequency, and treated acupoints; (4) a risk of bias assessment; and (5) outcome data, adverse effects, and drop-out rate.

EndNote will be used by the reviewers to facilitate data management and to identify duplicate publications. In cases where 2 or more reports describe the same trial, only one of these publications will be considered for inclusion in the analysis.

### Assessing Risk of Bias in Included Studies

The methodological quality of selected studies will be assessed using the Cochrane Risk of Bias 2.0 tool. Two reviewers (HX and CL) will independently assess the risk of bias for each included randomized controlled trial according to the guidelines provided in the *Cochrane Handbook for Systematic Reviews of Interventions* (version 6.3). The assessment will cover the following domains: (1) bias arising from the randomization process, (2) bias due to deviations from the intended intervention, (3) bias due to missing outcome data, (4) bias in measurement of the outcome, and (5) bias in selection of the reported results.

Each study will be classified as “low risk,” “high risk,” or “some concerns” based on the evaluation. Should any disagreements arise, a third reviewer (GL) will be engaged to facilitate consensus.

### Measures of Treatment Effect

Statistical analysis will be conducted using RevMan (version 5.3; Cochrane Collaboration), with forest plots being used to visually represent the comparative efficacy of the treatments. For continuous variables, the mean difference (MD) will be used to express the treatment effect, whereas for dichotomous data, the risk ratio (RR) will be used. For each treatment effect, the estimated value and 95% CIs will be determined and reported within the analysis.

### Dealing With Missing Data

For studies with missing or ambiguous data, efforts will be made to contact the first author or corresponding author for assistance. Should the missing data remain unobtainable despite attempts to contact the authors, the study will be excluded from the analysis.

For studies with missing data, the intention-to-treat analysis will be used to conduct statistical and omission analysis. Intention-to-treat analysis helps maintain the integrity of the original randomized allocation of participants, even in cases where some data may be missing. This approach guarantees that participants are evaluated based on their original randomized groups, irrespective of any missing data.

### Assessment of Reporting Biases

Should more than 10 studies report on the same outcome measure, a funnel plot and an Egger regression test will be applied to assess the potential for reporting bias.

### Data Analysis

RevMan will be used for the calculation of the RR for dichotomous data and the MD for continuous variables. For each effect, the estimated values and the 95% CIs will be determined.

The RR is calculated as follows:



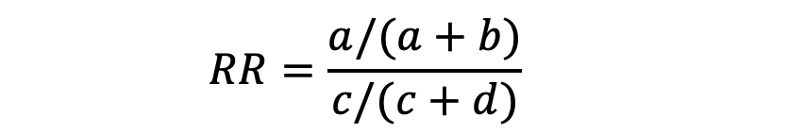



where *a* and *b* are the number of events and nonevents in the intervention group, and *c* and *d* are the number of events and nonevents in the control group.

The MD is calculated as follows:



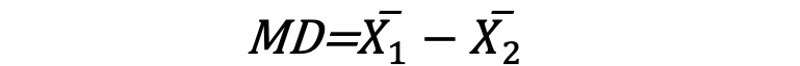



where 
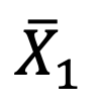
 and 
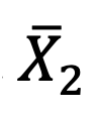
 are the means of the intervention and control groups, respectively.

Heterogeneity will be assessed using the *I*^2^ statistic, calculated as follows:



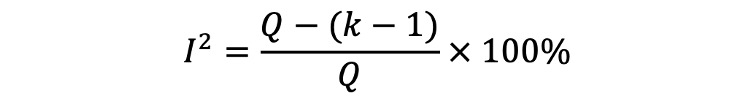



where *Q* is the Cochrane *Q* statistic and *k* is the number of studies.

If the research results exhibit low heterogeneity (*I*^2^<50%), a fixed-effect model will be applied for the meta-analysis. On the other hand, in case of significant heterogeneity (*I*^2^>50%), a random-effects model will be used for the meta-analysis, after which, further analysis will be conducted to ascertain the sources of heterogeneity.

When the data are not amenable to quantitative synthesis, a narrative summary will be provided to elucidate the findings of the included studies.

For trials that report only pre- and postintervention values, the mean change will be derived by computing the difference between post- and preintervention measurements. Consequently, the SD for these changes will be estimated.

### Subgroup Analysis

When significant heterogeneity is observed, a subgroup analysis will be performed to investigate the possible sources of this variability. The following factors will be examined as potential subgroup variables: age, gender, duration of COVID-19 infection, severity of insomnia, the type of acupuncture intervention, treatment frequency and duration, and other relevant parameters.

When sufficient data are available for each subgroup, a quantitative subgroup analysis will be performed to assess the influence of these factors on the treatment effects. Nonetheless, if the data for subgroup analysis are inadequate, a qualitative synthesis will be conducted. In this approach, the results from individual studies will be summarized and compared without a formal quantitative synthesis.

### Sensitivity Analysis

If significant heterogeneity persists even after the subgroup analysis, a sensitivity examination will be implemented. This process involves reconducting the meta-analysis after discarding studies of low quality or those with possible biased sources.

The comparison of the meta-analysis results before and after sensitivity analysis allows for a thorough assessment of the findings’ stability and reliability. This approach aids in understanding the impact of individual studies on the total results, offering a more in-depth insight into the meta-analysis outcomes.

### Grading the Quality of Evidence

Researchers will assess the quality of evidence using the Grading of Recommendations, Assessment, Development, and Evaluation (GRADE) approach. The quality of evidence will be classified into 4 levels: very low, low, moderate, or high.

The GRADE methodology facilitates a systematic and transparent assessment of the certainty and strength of the evidence, thereby enabling researchers to present a comprehensive and reliable summary of the evidence in a distinct and dependable way.

### Ethical Considerations

Ethics approval is not required since this protocol is solely for a systematic review and does not involve private data.

### Dissemination

The results will be disseminated electronically through publication in a peer-reviewed journal or presented at a relevant conference.

## Results

The study will be conducted following the methodology in the *Cochrane Handbook for Systematic Review of Interventions*. Two independent researchers will perform comprehensive searches across 12 databases, encompassing English databases (PubMed, Cochrane Library, Web of Science, Embase, OVID, and Scopus) and Chinese databases (China National Knowledge Infrastructure, Wan-Fang Data, the Chinese Biomedical Literature Database, the Chinese Scientific Journal Database, the Duxiu Database, and the Chinese Clinical Trial Registry Center). Focusing on the outcomes such as response rate, insomnia severity, TCMSS score, and adverse event rates, this study will deliver an evidence-based review of the efficacy and safety of acupuncture for PCI treatment.

## Discussion

### Overview

The COVID-19 pandemic has become one of the most formidable public health crises in recent years. As the persistence of post–COVID-19 condition continues, a considerable number of individuals have been grappling with PCI. This issue is escalating and poses a critical challenge to public health, placing a substantial economic burden on society. Not only does PCI negatively impact physical and mental well-being, but it also contributes to a decline in social functioning and a lower quality of life [20]. PCI is often accompanied by other symptoms, such as anxiety and depression [70]. Although CBT-i and pharmacological interventions are widely used in clinical settings, they face certain constraints, such as variable efficacy, adverse effects, and high costs. These limitations have sparked a surge of interest in CAM therapies.

As a significant component of CAM therapies, acupuncture has gained increasing recognition for its effectiveness, affordability, and favorable safety profile. It is rooted in the basic theory of traditional Chinese medicine, which emphasizes the interconnectedness of nature and humanity, viewing the body, mind, and spirit as a unified whole [71,72]. Considering that PCI affects both physical and mental health, acupuncture’s natural, potent, and low-risk attributes, along with its holistic approach, make it a compelling alternative for individuals with PCI. However, given that PCI is a relatively novel condition, it is imperative to conduct a thorough analysis of acupuncture’s therapeutic efficacy and safety as a treatment option.

This paper presents the protocol for a systematic review that constitutes the first quantitative analysis of the efficacy and safety of acupuncture for PCI. Through a meticulous evaluation of evidence gleaned from published randomized controlled trials, we aim to provide a comprehensive appraisal of acupuncture’s impact on PCI. Furthermore, the study endeavors to delineate the optimal modality, frequency of intervention, and duration of treatment to achieve the most favorable outcomes. The findings from this meta-analysis and systematic review are expected to offer valuable insights to clinical practitioners, establishing a robust theoretical foundation and essential reference for the application of CAM therapies in PCI treatment. This evidence may significantly enhance the quality of informed clinical decision-making, particularly in the selection of therapeutic strategies for patients with PCI.

All amendments to this protocol will be meticulously recorded, including the amendment date, a description of the changes, and the corresponding rationale.

### Limitations

Due to language barriers, some relevant electronic databases might not be included, potentially limiting the comprehensiveness of the findings.

### Conclusion

This review will offer a thorough synthesis of the literature on acupuncture as a treatment for PCI, thereby enriching the current scientific discourse on this subject. By examining the evidence base, the review will deliver significant insights aimed at optimizing clinical practices in practical health care environments. It will also present health care professionals with a novel viewpoint on the use of complementary and alternative medicine in addressing PCI, facilitating more informed clinical decision-making.

## Appendix

Multimedia Appendix 1 Search strategy in PubMed database.

Multimedia Appendix 2 Data extraction tool.

## Abbreviations

CAM: complementary and alternative medicine
CBT-i: cognitive-behavioral therapy for insomnia
GRADE: Grading of Recommendations, Assessment, Development, and Evaluation
MD: mean difference
PCI: post–COVID-19 insomnia
PRISMA: Preferred Reporting Items for Systematic Reviews and Meta-Analyses
RR: risk ratio
TCMSS: Traditional Chinese Medicine Symptom Scale
