# Supplementary material for: Efficacy and Safety of Acupuncture for Post–COVID-19 Insomnia: Protocol for a Systematic Review and Meta-Analysis
Source: JMIR Res Protoc. 2025 Mar 3;14:e69417. doi: 10.2196/69417 (PMC11914848; doi:10.2196/69417)
Supplement: Multimedia Appendix 1 [file resprot_v14i1e69417_app1.docx]

**Appendix 1.** Search strategy in PubMed database.

| **Search step** | **Search query** |
| --- | --- |
| #1 | "COVID-19"[MeSH] |
| #2 | "2019-nCoV Infection"[Tiab] OR "2019 nCoV Infection"[Tiab] OR "2019-nCoV Infections"[Tiab] OR "Infection, 2019-nCoV"[Tiab] OR "SARS-CoV-2 Infection"[Tiab] OR "Infection, SARS-CoV-2"[Tiab] OR "SARS CoV 2 Infection"[Tiab] OR "SARS-CoV-2 Infections "[Tiab] OR "Infection, 2019-nCoV"[Tiab] OR "2019 Novel Coronavirus Disease"[Tiab] OR "2019 Novel Coronavirus Infection"[Tiab] OR "2019-nCoV Disease"[Tiab] OR "2019 nCoV Disease"[Tiab] OR "2019-nCoV Diseases"[Tiab] OR " Disease, 2019-nCoV"[Tiab] OR "COVID19"[Tiab] OR " COVID 19"[Tiab] OR " Coronavirus Disease 2019"[Tiab] OR "Disease 2019, Coronavirus"[Tiab] OR "Coronavirus Disease-19"[Tiab] OR " Coronavirus Disease 19"[Tiab] OR "Severe Acute Respiratory Syndrome Coronavirus 2 Infection"[Tiab] OR "COVID-19 Virus Disease "[Tiab] OR "COVID 19 Virus Disease"[Tiab] OR "COVID-19 Virus Diseases"[Tiab] OR "Disease, COVID-19 Virus "[Tiab] OR "Virus Disease, COVID-19"[Tiab] OR " SARS Coronavirus 2 Infection"[Tiab] OR "COVID-19 Virus Infection"[Tiab] OR "COVID 19 Virus Infection"[Tiab] OR "COVID-19 Virus Infections"[Tiab] OR "Infection, COVID-19 Virus"[Tiab] OR "Virus Infection, COVID-19"[Tiab] OR "COVID-19 Pandemic"[Tiab] OR "COVID 19 Pandemic"[Tiab] OR "Pandemic, COVID-19"[Tiab] OR "COVID-19 Pandemics"[Tiab] |
| #3 | #1 OR #2 |
| #4 | "Acupuncture Therapy"[MeSH] |
| #5 | "Acupuncture Treatment"[Tiab] OR "Acupuncture Treatments"[Tiab] OR "Treatment, Acupuncture"[Tiab] OR "Therapy, Acupuncture"[Tiab] OR “Pharmacoacupuncture Treatment "[Tiab] OR "Treatment, Pharmacoacupuncture"[Tiab] OR "Pharmacoacupuncture Therapy"[Tiab] OR “Therapy, Pharmacoacupuncture"[Tiab] OR "Acupotomy"[Tiab] OR “Acupotomies"[Tiab] |
| #6 | #4 OR #5 |
| #7 | "Sleep Initiation and Maintenance Disorders"[MeSH] |
| #8 | "DIMS (Disorders of Initiating and Maintaining Sleep)"[Tiab] OR " Disorders of Initiating and Maintaining Sleep”[Tiab] OR "Sleeplessness"[Tiab] OR "Insomnia Disorder"[Tiab] OR “Insomnia Disorders"[Tiab] OR "Insomnia"[Tiab] OR "Insomnias"[Tiab] OR “Chronic Insomnia"[Tiab] OR "Insomnia, Chronic”[Tiab] OR “Early Awakening"[Tiab] OR “Awakening, Early"[Tiab] OR "Nonorganic Insomnia”[Tiab] OR “Insomnia, Nonorganic"[Tiab] OR “Primary Insomnia"[Tiab] OR "Insomnia, Primary”[Tiab] OR “Psychophysiological Insomnia"[Tiab] OR “Insomnia, Psychophysiological"[Tiab] OR “Rebound Insomnia"[Tiab] OR "Insomnia, Rebound”[Tiab] OR “Secondary Insomnia"[Tiab] OR “Insomnia, Secondary"[Tiab] OR "Sleep Initiation Dysfunction”[Tiab] OR “Dysfunction, Sleep Initiation"[Tiab] OR “Dysfunctions, Sleep Initiation"[Tiab] OR “Sleep Initiation Dysfunctions"[Tiab] OR "Transient Insomnia”[Tiab] OR “Insomnia, Transient"[Tiab] |
| #9 | #7 OR #8 |
| #10 | "randomized controlled trial"[Publication Type] OR "randomized"[Tiab] OR "placebo"[Tiab] |
| #11 | #3 AND #6 AND #9 AND #10 |
