# Supplementary material for: Efficacy and Safety of Acupuncture for Post–COVID-19 Insomnia: Protocol for a Systematic Review and Meta-Analysis
Source: JMIR Res Protoc. 2025 Mar 3;14:e69417. doi: 10.2196/69417 (PMC11914848; doi:10.2196/69417)
Supplement: Multimedia Appendix 2 [file resprot_v14i1e69417_app2.docx]

**Appendix 2:** Data Extraction Tool

| Author and Date | Country | Sample size | Diagnosis for Inclusion | Severity of insomnia (mild/middle/severe) | Patients age | course of disease | Blinding | Intervention | Treatment frequency (treatment period) | Treated acupoints | Outcome measures | Adverse effects | Drop-out rate |
| --- | --- | --- | --- | --- | --- | --- | --- | --- | --- | --- | --- | --- | --- |
|  |  |  |  |  |  |  |  |  |  |  |  |  |  |
|  |  |  |  |  |  |  |  |  |  |  |  |  |  |
|  |  |  |  |  |  |  |  |  |  |  |  |  |  |
|  |  |  |  |  |  |  |  |  |  |  |  |  |  |
